# Supplementary material for: Chromothripsis during telomere crisis is independent of NHEJ, and consistent with a replicative origin
Source: Genome Res. 2019 May;29(5):737–49. doi: 10.1101/gr.240705.118 (PMC6499312; doi:10.1101/gr.240705.118)
Supplement: Supplemental Material [file supp_gr.240705.118_Supplemental_file_1.zip › contigs/annotated_contigs/DB38/contig.2.DB38_length_495_mean_cov_5.53333333333.docx]

**DB38_length_495_mean_cov_5.53333333333**

```
CTTCTTCTCAGATCTCTCTTTTCTAAACTTTAAATGCAGCTGCTTAGTGAACCTAAGCACTCGTGTATCTCACAAGCACCTCAAATTCA
 >chr4:26678589-26678873 + E=2e-159
AGATGTCAACACTCTTCCTAAATTCCTTTTTCAGTACCACTGTCCATCAAGGAACCGTAGCCAGAAACCTGGTGTCATCCCTAGCTCCT

GAATATCATATTGTATTACTCGATAATTATACTACTCAATAATTCCTTCTTGATTTAACTCCTAAACATGTTTCACTACCACTCTCATT

TCAAAATACCAT|CATTT|TTTGTGCTATTTCAATTAAAGCCATTTTTCAATATTTTAGGTTTAATGAAAATACTAAAATATTCCAGTC
             >chr4:26385861-26386077 + E=2e-118
ACAGATACTTAATTGCTTGTGAATTATCAATTCATAAACAGTATCTTAGGACAAATGAACAAAATGAACTAAATGGGATCCTATGACTT

TTATCATCAAAGTTAATAATCTTGTGGAACATTACAAATACAGTAGAGAAAT
```
